# Substrate-driven reprogramming of the rhizosphere metabolome underlies enhanced tomato growth and quality in soilless cultivation

**DOI:** 10.3389/fpls.2026.1783719

**Published:** 2026-04-01

**Authors:** Yu Chen, Yuyuan Chai, Xi Chen, Jing Shi

**Affiliations:** 1 College of Resources and Environment, Yunnan Agricultural University, Kunming, China; 2 Key Laboratory for Improving Quality and Productivity of Arable Land of Yunnan Province, College of Resources and Environment, Yunnan Agricultural University, Kunming, Yunnan, China; 3 Kunming Changshui International Airport Power Energy Department, Kunming, China

**Keywords:** culture medium, metabolomics, peat substrate, pinecone residue substrate, rhizosphere metabolites

## Abstract

**Introduction:**

The rhizosphere metabolome is a crucial mediator of plant-substrate interactions, yet how different cultivation substrates reprogram this metabolic interface and influence crop performance remains poorly understood.

**Methods:**

Using tomato as a model, we employed non-targeted metabolomics based on solvent extraction followed by gas chromatography-mass spectrometry (GC-MS) to compare rhizosphere metabolic profiles under three substrates: conventional facility soil (SL), pinecone residue (PR), and peat substrate (PS).

**Results:**

We identified and annotated 276 metabolites, with lipids and lipid-like molecules being the most abundant class (24.28%). Substrate type fundamentally reshaped the metabolome, with PR inducing the most distinct profile and PS triggering the most extensive metabolic reprogramming (277 differential metabolites). Notably, over 91% of differential metabolites were up-regulated upon tomato cultivation. The superior physicochemical properties (e.g., lower bulk density, higher nutrient availability) of organic substrates (PR and PS) were linked to both the distinct metabolic signatures and significant enhancements in tomato growth and fruit quality, particularly in PS which showed an 80.08% yield increase and a 50.19% boost in fruit vitamin C content.

**Discussion:**

Our findings demonstrate that organic substrates, especially peat, enhance plant performance by activating a more diverse and functionally specialized rhizosphere metabolome, providing a mechanistic basis for optimizing substrate selection in facility agriculture.

## 1 Introduction

The shift from traditional soil-based to soilless cultivation systems in facility agriculture has become a pivotal strategy for overcoming soil degradation challenges, such as acidification, nutrient imbalance, and the buildup of soil-borne pathogens after continuous cropping (Gruda, 2019). In these systems, the growth substrate is not merely a physical anchor but the primary environment governing root development, nutrient access, and ultimately, crop productivity and quality (Putra and Yuliando, 2015). The rhizosphere, the narrow zone of soil influenced by plant roots, is the critical interface where these substrate effects are mediated. Root exudates, sloughed-off cells, as well as metabolites produced by the active rhizosphere microbiota, collectively form a diverse rhizosphere metabolome, comprising sugars, organic acids, phenolics, and other secondary metabolites (Bais et al., 2006; Liu et al., 2020). This chemical milieu plays a fundamental role in shaping microbial communities, driving nutrient cycling, and modulating plant health and stress responses (Hu et al., 2018; Wen et al., 2020). Consequently, the physicochemical properties of the cultivation substrate, such as bulk density, porosity, pH, water-holding capacity, and nutrient availability, exert a profound influence on root morphology and the composition of root exudates, thereby reprogramming the rhizosphere metabolic environment (Jones et al., 2004; Berg and Smalla, 2009). Comparative studies have demonstrated distinct microbial communities and rhizosphere chemistry in plants grown in mineral versus organic substrates, underscoring the substrate’s role as a key driver of rhizosphere processes (Grunert et al., 2016; Miller et al., 2019).

Despite this recognition, a critical knowledge gap persists. While previous research has often focused on either substrate properties or microbial community shifts, a systematic understanding of how specific substrate types reconfigure the rhizosphere metabolome and how these metabolic changes subsequently link to measurable improvements in plant growth and fruit quality remains elusive. This is particularly true for novel, sustainable organic substrates like pinecone residue, whose effects on rhizosphere metabolism are largely unknown. Addressing this gap requires a tool capable of capturing the broad spectrum of rhizosphere chemicals. Non-targeted metabolomics, leveraging techniques like gas chromatography-mass spectrometry (GC-MS), offers a powerful solution by enabling the simultaneous detection and annotation of hundreds to thousands of metabolites, providing a holistic view of metabolic responses (Zhao et al., [[NoYear]]; Li et al., 2025).

Tomato (Solanum lycopersicum L.) is a globally important vegetable and a model crop for facility production, making it an ideal subject for such an investigation (Gruda, 2019). This study was designed to explore associations between substrate properties, rhizosphere metabolism, and plant performance. We conducted a pot experiment comparing a conventional facility soil (SL) with two organic substrates: pinecone residue substrate (PR) and peat substrate (PS). We hypothesized that organic substrates would induce distinct rhizosphere metabolic profiles correlated with improved tomato growth and fruit quality, while acknowledging that substrate nutrient status may also directly contribute to these phenotypic differences. To test this, we first characterized the substrates’ physicochemical properties. We then employed GC-MS-based non-targeted metabolomics to profile the rhizosphere metabolome and integrated these data with comprehensive measurements of tomato agronomic and quality traits. By bridging substrate science with rhizosphere metabolomics, this work aims to provide a mechanistic basis for selecting and optimizing cultivation substrates, thereby contributing to the development of more productive and sustainable soilless production systems.

## 2 Materials and methods

### 2.1 Test design and plant cultivation

The experimental plant was tomato (*Solanum lycopersicum* L. cv. ‘22B’). Three growing media were used: facility soil (SL), a typical Yunnan plateau red soil collected from the 0–20 cm surface layer of a tomato greenhouse at Yunnan Agricultural University; pinecone residue substrate (PR), produced by crushing and composting pinecones (Dali Yunfei Agricultural Science and Technology Co., Ltd.); and peat substrate (PS), consisting of imported white peat (PSM710) mixed with domestic peat at a 0.9:1 volume ratio, amended with perlite (perlite:substrate = 1:15, v/v). Their basic physicochemical properties are summarized in **Table 1** (analysis conducted by Yunnan Cloud Measurement Quality Inspection Co., Ltd.). Seeds were sown in a 50-cell tray on April 5, 2023. After 40 days, uniform seedlings were transplanted individually into fabric pots (35 cm diameter × 30 cm height) filled with approximately 0.02 m³ of the respective substrate to a depth of 20–25 cm. Following a 7-day equilibration period, 80 g of controlled-release compound fertilizer was incorporated into each pot as basal nutrition. To minimize confounding variables associated with post-transplant nutrient inputs and to maintain ecological relevance, a uniform basal fertilization regime was implemented across all treatment groups, whereby a single application of Basacote^®^ Plus 6M—a fully coated, controlled-release compound fertilizer providing nitrogen, phosphorus, potassium, and a full range of micronutrients with a nutrient release duration of six months—was incorporated into each substrate at a rate of 2 kg/m³ at the time of planting, and no additional fertilization was applied after transplanting. Due to inherent variations in the initial nutrient status of the substrates (**Table 1**), the total nutrient supply differed among treatments, and although this experimental setup reflects common horticultural practice, it introduces a methodological limitation, as it does not allow for the complete separation of direct nutrient effects from substrate-induced metabolic changes in plants—an issue addressed in detail in the Discussion section. The experiment included three cultivated treatments (SL, PR, and PS), each comprising 34 plants (totaling 102 plants), and three non-cultivated control groups (SLWP, PRWP, and PSWP), each with six replications, with all control plots receiving the same irrigation and fertilization protocols as the cultivated treatments. Pest and disease management followed standard integrated practices, with foliar applications of emamectin benzoate·chlorfenapyr, thiamethoxam·lambda-cyhalothrin, and pyraclostrobin·tebuconazole carried out as needed strictly in accordance with the manufacturers’ guidelines, and all pesticide applications were scheduled with a minimum interval of seven days before or after any plant sampling event to avoid interference with experimental measurements. Detailed information on the experimental procedures, including application timing, dosages, and monitoring protocols, is provided in **Supplementary Table 1** of the Supplementary Material.

**Table 1:** Basic physicochemical properties of tested soil and cultivation substrates.

Index	Soil	Pinecone residue substrate	Peat substrate
pH	6.38	6.54	6.41
EC (mS·cm^-1^)	1.670	0.610	1.125
SOM (g·kg^-1^)	27.5	559.0	616.0
TN (%)	0.192	0.696	1.078
AP (mg·kg^-1^)	29.9	169.2	62.0
AK (mg·kg^-1^)	1054	2223	1882
E-Ca^2+^ (mol·kg^-1^)	13.4	36.6	72.2
E-Mg^2+^ (mol·kg^-1^)	3.6	11.0	21.0
E-K^+^ mol·kg^-1^)	2.65	5.34	5.37
E-Na^+^ (mol·kg^-1^)	0.54	0.45	0.91

EC, electric conductivity; SOM, soil organic matter; TN, total nitrogen; AP, available phosphorus; AK, available potassium; E-Ca2+, exchangeable calcium; E-Mg2+, exchangeable magnesium; E-K+, exchangeable potassium; E-Na+, exchangeable sodium. Although both AK and E-K^+^ characterize the potassium forms available to plants, the simultaneous determination of both can provide a more comprehensive assessment of potassium availability. In addition, analyzing the exchangative acon spectrum is crucial for evaluating the synergistic or antagonistic interactions among ions, which directly affect the nutrient absorption and metabolic processes of plants.

### 2.2 Sampling and determination methods

Non-rhizosphere soil/substrate sampling: During tomato harvest, non-rhizosphere soil and substrate samples were collected from potted plants using a sterilized ring knife (100 cm³ volume). Triplicate samples per treatment were randomly collected to ensure statistical robustness. Immediately after collection, samples were weighed for bulk density calculation and gravimetrically determined moisture content. Subsequently, samples were air-dried at ambient temperature, sieved through a 50-mesh screen, and stored desiccated at room temperature for physicochemical characterization.

Rhizosphere soil/substrate samples: at the 16th week post-transplantation of tomato plants, select healthy specimens and carefully excavate the underground portions while striving to preserve the integrity of the root system. Subsequently, gently remove larger clumps of soil or substrate from the roots until no substantial material remains. Finally, use a gentle shake and a soft-bristled brush to carefully remove any remaining soil or substrate, and evenly collect rhizosphere samples of fibrous roots and main roots. Six samples were randomly collected from each treatment for statistical repetition. Rhizosphere samples were stored at -80 °C refrigerator for GC-MS (gas chromatography-mass spectrometry) non-target metabolomics detection.

Shanghai Baiqu Biomedical Technology Co., Ltd. was entrusted to perform GC-MS non-target metabolomics detection on soil and substrate rhizosphere samples. The main instruments are shown in **Supplementary Table 2** (Which is in the Supplementary materials).

Soil moisture content (%) was determined by measuring the mass difference after drying the soil at 105°C until constant weight was achieved. Soil pH and electrical conductivity (EC) were measured following the methods described in Ji et al (Ji et al., 2021). Total nitrogen content (TN) was analyzed using the dry combustion method with an elemental analyzer (Vario MACRO cube, Elementar, Germany). Soil organic carbon (SOC) was determined via wet digestion using the K_2_Cr_2_O_7_ oxidation method, as detailed in Wang et al (Wang et al., 2016). Ammonium nitrogen concentration was determined with the indophenol blue colourimetric method (Dorich and Nelson, 1983), while nitrate nitrogen was determined using dual-wavelength ultraviolet spectrophotometry (Norman et al., 1985). Available phosphorus (AP) was extracted with sodium bicarbonate according to Shigaki and Sharpley (2011), and available potassium concentration (AK) was determined with a flame photometer after extraction with ammonium acetate (NH_4_AC) (Lu et al., 2017).

At harvest, the following morphological and physiological parameters were quantified: plant height, stem diameter, fresh weight of roots, stems, leaves, and fruits, total fresh weight, root length, root-shoot ratio, and root vitality index in rhizosphere soil/substrate samples. Root vitality, expressed as dehydrogenase (DHA) activity in fresh roots, was quantified using the dehydrogenase (DHA) assay kit (Kit No. YX-W-C109) from Shanghai Youxuan Biotechnology Co., Ltd. Tomato fruit quality parameters were evaluated at harvest. Vitamin C (ascorbic acid) content was determined by high-performance liquid chromatography (HPLC). Soluble solids content was measured using a handheld refractometer (CSOIF WAY-80, Shanghai Optical Instrument Co.). Sugar content, titratable acidity, and sugar-to-acid ratio were analyzed using a digital saccharimeter and acidity meter (PAL-BX/ACID, APTO, Japan). Citric acid content was determined using a citric acid (CA) assay kit from Shanghai Youxuan Biotechnology Co., Ltd. Lycopene content was quantified using a plant lycopene ELISA detection kit.

Metabolites Extraction: 500 mg sample was transferred into a 5 mL tube, and 1 mL pre-cold extraction mixture (methanol/dH_2_O (v:v) =3:1) and 1 mL Ethyl acetate with internal standard (adonitol, 0.5mg/mL stock), were added. Samples were vortexed for 30 s and homogenized with ball mill for 4 min at 40 Hz, followed by ultrasonication for 5 min in ice water(repeat 3 time). After centrifugation at 4 °C for 15 min at 10000 rpm (RCF = 9600 (×*g*), R = 8.6 cm), transfer supernatant into the 5 mL EP tubes. Add 1 mL pre-cold extraction mixture (methanol/dH_2_O (v:v) =3:1) and 1 mL EA, repeat steps 2 and 3 and combine all the liquid supernatants. 2 mL supernatant was transferred to a fresh tube To prepare the QC (Quality control) sample, 650 μL of each sample was taken out and combinedtogether. After evaporation in a vacuum concentrator, 30 μL of Methoxyamination hydrochloride (20 mg/mL in pyridine) was added and then incubated at 80 °C for 30 min, then derivatized by 40 μL of BSTFA regent (1% TMCS, v/v) at 70 °C for 1.5 h. Gradually cooling samples to room temperature, 5 μL of saturated Fatty Acid Methyl Ester (FAMEs) (in chloroform) was added to QC sample. All samples were then analyzed by Gas Chromatography Tandem Time-of-Flight Mass Spectrometer (GC-TOFMS) (The brands and origins of the reagents used in the experiment are shown in **Supplementary Table 2**).

Gas Chromatography Tandem Time-of-Flight Mass Spectrometer (GC-TOFMS) Analysis: GC-TOFMS analysis was performed using an SHIMADZUGC-2020 gas chromatograph coupled with a time-of-flight mass spectrometer. The system utilized a DB-5MS capillary column (30m×250μm×0.25μm, J&W Scientific, Folsom, CA, USA),The specific analysis conditions of GC-MS are as follows **Supplementary Table 3**. 1 μL aliquot of sample was injected in splitless mode. Helium was used as the carrier gas, the front inlet purge flow was 3 mL min^−1^, and the gas flow rate through the column was 1 mL min^−1^. The initial temperature was kept at 50 °C for 1 min, then raised to 310 °C at a rate of 8 °C min^−1^, then kept for 11.5 min at 310 °C. The injection, transfer line, and ion source temperatures were 280, 280 and 200 °C, respectively. The energy was -70 eV in electron impact mode. The mass spectrometry data were acquired in full-scan mode with the m/z range of 50–500 at a rate of 12.5 spectra per second after a solvent delay of 7.2 min (The specific parameters of the instruments used in the experiment are shown in **Supplementary Table 4**).

### 2.3 Data processing

Microsoft Excel for Mac (V 16.80) was used to sort out all the original data of the experiment. Data were analyzed using IBM SPSS (V 28.0). The mean ± standard error (Mean ± SE) is used to describe the statistical values in the data graph, and lowercase letters are used to indicate significant differences between the data. Raw data analysis, including peak extraction, baseline adjustment, deconvolution, alignment and integration was finished with Chroma TOF (V 4.3x, LECO) software and LECO-Fiehn Rtx5 database was used for metabolite identification by matching the mass spectrum and retention index. Finally, the peaks detected in less than half of QC samples or RSD>30% (the RSD of the QC samples was 8.09%) in QC samples was removed (Dunn et al., 2011). The raw data for GC-MS metabolite detection included 5 quality control (QC) samples and 36 test samples (each treatment was repeated 6 times), and a total of 1117 peaks were detected. After quality and reliability screening, 848 peaks were retained for further analysis. In this study, the observed superimposition of retention times and peak areas across QC TIC profiles confirmed robust instrumental stability during analysis (**Supplementary Figure 1**). The LECO-Fiehn Rtx5 database was used in the qualitative phase of the material, including mass spectrometry matching and retention time index matching. For metabolomics data, p-values were calculated using Student’s t-test (two-tailed, unequal variance) for pairwise comparisons. To control for false discovery, Benjamini–Hochberg correction was applied (q < 0.05). Metabolites with variable importance in projection (VIP) > 1 from orthogonal partial least squares-discriminant analysis (OPLS-DA) models and p < 0.05 were considered differentially accumulated. OPLS-DA model validity was assessed using Q² values (all > 0.59) and permutation testing (n = 200). Principal component analysis (PCA) and OPLS-DA were performed using SIMCA software (v16.0.2). Hierarchical clustering analysis (HCA) was performed using the pheatmap package in R (v4.1.0). Venn diagrams were generated using the Venny 2.1 online tool. KEGG pathway enrichment analysis was conducted using MetaboAnalyst 5.0.

## 3 Results

### 3.1 The physicochemical properties of various cultivation substrates and their influence on tomato growth, yield, and quality

The physicochemical properties of the cultivation media differed markedly at harvest (**Table 2**). Compared to facility soil treatments (SL, SLWP), the organic substrates (PR, PS) exhibited significantly lower bulk density, higher moisture content, and elevated levels of ammonium nitrogen (NH_4_^+^-N), nitrate nitrogen (NO_3_^-^-N), available phosphorus (AP), available potassium (AK), and soil organic matter (SOM). Specifically, the PS treatment showed the highest electrical conductivity (1.30 mS·cm^-^¹) and the lowest pH (5.00). These favorable substrate conditions were associated with superior tomato performance (**Tables 3**, **4**). Plants grown in PS substrate achieved the highest values in plant height, root length, fresh biomass of all organs, and total yield (80.08% higher than SL). While PR treatment also improved growth metrics relative to SL, the enhancements were generally more pronounced under PS cultivation. Fruit quality was significantly modulated by substrate type (**Table 5**). Although SL-grown fruits had the highest sugar and titratable acidity, their sugar-to-acid ratio (8.44) was suboptimal for flavor. In contrast, both PR and PS treatments yielded fruits with balanced sugar-to-acid ratios (10.93 and 11.34, respectively), alongside significantly higher soluble solids and vitamin C content, indicating improved nutritional and sensory quality. Notably, the higher vitamin C content in PS-grown fruits was positively correlated with elevated available potassium levels in the substrate, which is consistent with potassium’s known role in activating antioxidant metabolism in plants (Khan et al., 2023; Siddiqui et al., 2024).

**Table 2:** Physical and chemical properties of cultivation media in different treatments of tomato harvesting period.

Treatment	SLWP	PRWP	PSWP	SL	PR	PS
Volumetric weight (kg·m^-3^)	920.58 ± 8.05 a	147.43 ± 3.93 d	110.65 ± 2.31 e	878.90 ± 29.13 b	185.71 ± 12.41 c	101.08 ± 7.91 e
Moisture content (%)	0.44 ± 0.04 c	1.69 ± 0.17 b	2.56 ± 0.10 a	0.46 ± 0.08 c	1.57 ± 0.10 b	2.12 ± 0.12 b
pH value	5.56 ± 0.18 b	5.89 ± 0.27 a	5.39 ± 0.16 b	5.93 ± 0.14 a	5.47 ± 0.08 b	5.00 ± 0.05 c
Electrical Conductivity (mS·cm^-1^)	0.77 ± 0.08 bc	0.65 ± 0.03 bc	0.81 ± 0.17 b	0.61 ± 0.09 c	0.79 ± 0.02 b	1.30 ± 0.08 a
Ammonium Nitrogen (mg·kg^-1^)	19.26 ± 1.56 f	63.65 ± 4.28 d	96.62 ± 3.28 c	40.31 ± 2.79 e	262.20 ± 0.11 a	181.21 ± 11.16 b
Nitrate Nitrogen (mg·kg^-1^)	62.20 ± 8.00 d	57.91 ± 9.46 d	133.29 ± 13.57 b	64.45 ± 5.72 d	199.44 ± 10.83 a	111.32 ± 5.07 c
Available Phosphorus (mg·kg^-1^)	232.21 ± 10.80 d	361.81 ± 22.82 b	321.39 ± 14.77 c	247.79 ± 20.59 d	402.23 ± 23.52 a	302.54 ± 14.89 c
Available Potassium (mg·kg^-1^)	256.42 ± 27.81 d	422.69 ± 8.17 b	429.50 ± 3.19 b	349.84 ± 22.87 c	504.03 ± 10.72 a	513.93 ± 26.59 a
Soil Organic Matter (mg·kg^-1^)	38.21 ± 16.85 c	128.73 ± 11.07 b	155.62 ± 7.28 a	21.77 ± 2.18 d	133.64 ± 3.60 b	161.42 ± 2.05 a

The data are mean ± standard error. Different letters in the same column indicated that the parameter was significantly different between different treatments, using one-way analysis of variance, *P* < 0.05.

**Table 3:** Tomato growth and yield under substrate and soil cultivation conditions.

Treatments	Plant height (cm)	Stem diameter (mm)	Fresh root weight (kg)	Fresh stem weight (kg)	Fresh leaves weight (kg)	Fresh fruit weight (kg)	Total fresh weight (kg)	Estimated output value (kg/667m^2^)
SL	196.11 ± 37.00 b	13.08 ± 0.67 a	0.10 ± 0.01 b	0.20 ± 0.01 b	0.43 ± 0.03 c	1.89 ± 0.20 b	2.62 ± 0.21 b	1986.43 ± 158.72 c
PR	227.56 ± 35.08 a	14.81 ± 1.74 a	0.11 ± 0.01 ab	0.23 ± 0.02 b	0.48 ± 0.01 b	2.04 ± 0.16 b	2.86 ± 0.18 b	2561.67 ± 218.14 b
PS	252.49 ± 28.64 a	14.86 ± 0.78 a	0.12 ± 0.01 a	0.31 ± 0.03 a	0.54 ± 0.01 a	2.58 ± 0.22 a	3.56 ± 0.26 a	3577.18 ± 242.04 a

The data are mean ± standard error. Different letters in the same column indicated that the parameter was significantly different between different treatments, using one-way analysis of variance, *P* < 0.05.

**Table 4:** Root activity of tomatoes under substrate and soil cultivation conditions.

Treatments	Root length (cm)	Root-shoot ratio	Root vitality (mg/d/g)
SL	60.37 ± 0.81 b	0.042 ± 0.004 a	5.03 ± 0.48 a
PR	75.00 ± 3.67 a	0.039 ± 0.005 a	4.93 ± 0.34 a
PS	72.87 ± 3.01 a	0.035 ± 0.001 a	5.50 ± 0.38 a

The data are mean ± standard error. Different letters in the same column indicated that the parameter was significantly different between different treatments, using one-way analysis of variance, *P* < 0.05.

**Table 5:** Tomato quality under substrate-based and soil cultivations.

Treatment	Sugar content (%)	Titratable acid (%)	Sugar acid ratio	Soluble solid content(%)	Vitamin C (mg/kg)	Citric acid (mg/kg)	Lycopene (mg/kg)
SL	5.80 ± 0.44 a	0.69 ± 0.07 a	8.44 ± 0.77 b	4.44 ± 0.40 b	81.31 ± 0.63 b	17.13 ± 4.78 a	21.61 ± 2.16 a
PR	5.30 ± 0.26 a	0.49 ± 0.05 b	10.93 ± 0.60 a	5.10 ± 0.18 a	96.20 ± 4.16 b	22.50 ± 4.25 a	24.24 ± 1.31 a
PS	4.23 ± 0.25 b	0.37 ± 0.02 c	11.34 ± 0.26 a	5.49 ± 0.46 a	122.12 ± 15.5 a	18.00 ± 3.55 a	22.79 ± 2.64 a

The data are mean ± standard error. Different letters in the same column indicated that the parameter was significantly different between different treatments, using one-way analysis of variance, *P* < 0.05.

### 3.2 Composition and classification of rhizosphere metabolites

**Figure 1:**
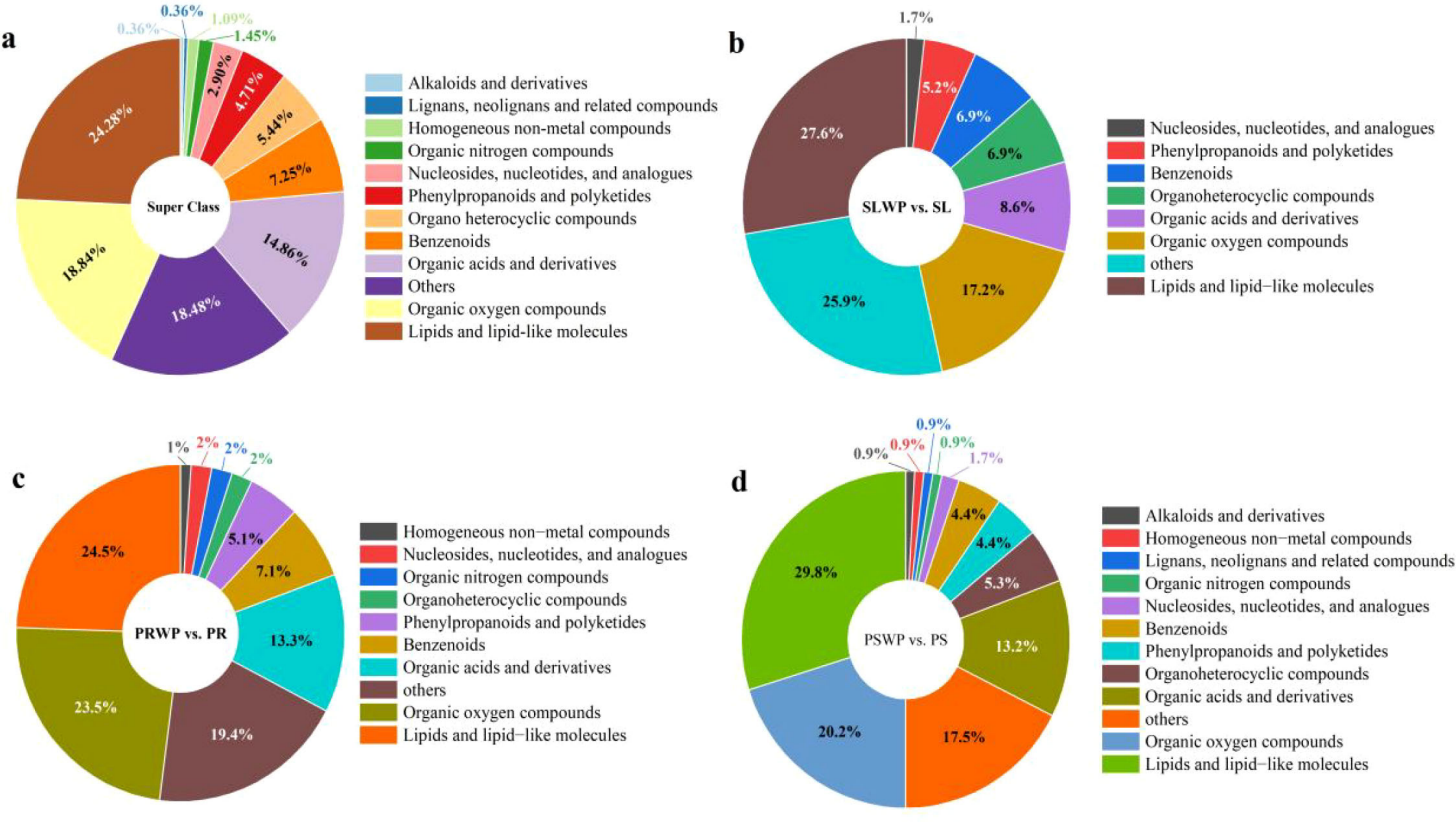
Secondary classification annotation of metabolites in tomato rhizosphere. **Figure 1** presents the secondary classification annotations of differentially accumulated metabolites across four comparative analyses, organized into four distinct **(a-d)**. **(a)** displays the overall secondary classification profile of all differentially accumulated metabolites identified across the entire dataset. **(b–d)** respectively illustrate the secondary classification distributions specific to the SLWP vs. SL, PRWP vs. PR, and PSWP vs. PS comparison groups. In each panel, the “Others” category encompasses metabolite classes not individually represented in that particular visualization; the composition of this category is therefore group-specific and reflects the residual classifications after exclusion of the major annotated classes.

To characterize metabolite variation across different cultivation substrates, primary metabolites in the samples were identified using gas chromatography-mass spectrometry (GC-MS) non-targeted metabolomics. For each treatment (including planted and unplanted control groups), six independent biological replicates were analyzed, ensuring robust statistical power for multivariate modeling. A total of 848 metabolites were identified in this experiment, and the relevant data was queried and organized using HMDB and KEGG databases. After the removal of 285 unidentified substances and 287 duplicate substances, a total of 276 metabolites included in the database were ultimately obtained. They can be categorized into 12 secondary classifications (Super Class), as shown in **Figure 1**. Lipids and lipid-like molecules constituted the most abundant category (24.28%), followed by Organic oxygen compounds (18.84%) and Organic acids and derivatives (14.86%). In contrast, lignans and alkaloids were minor components (0.36% each), suggesting a metabolic profile centered on membrane constituents, energy metabolism, and organic acid dynamics. The super class composition of differential metabolites in the tomato rhizosphere before and after cultivation across different substrates exhibited significant variation (**Figures 1b–d**). The comparison between PSWP and PS (peat substrate) displayed the highest metabolite diversity, encompassing a total of 12 super classes. Notably, this group uniquely contained the compound classes “alkaloids and their derivatives” and “lignans, neolignans, and related compounds,” which are strongly associated with plant defense mechanisms and stress responses. These compound classes were either undetected or present in negligible proportions in all other comparison groups. Furthermore, “lipids and lipid-like molecules” constituted the most abundant category across all groups, whereas the levels of “organic acids and their derivatives” in the PS and PR groups were significantly higher compared to those in the SL group. These findings suggest that the peat substrate induces more diverse and functionally specialized metabolic responses in the tomato rhizosphere, particularly in secondary metabolism related to defense.

### 3.3 The overall difference of rhizosphere metabolites

#### 3.3.1 Cluster analysis

**Figure 2:**
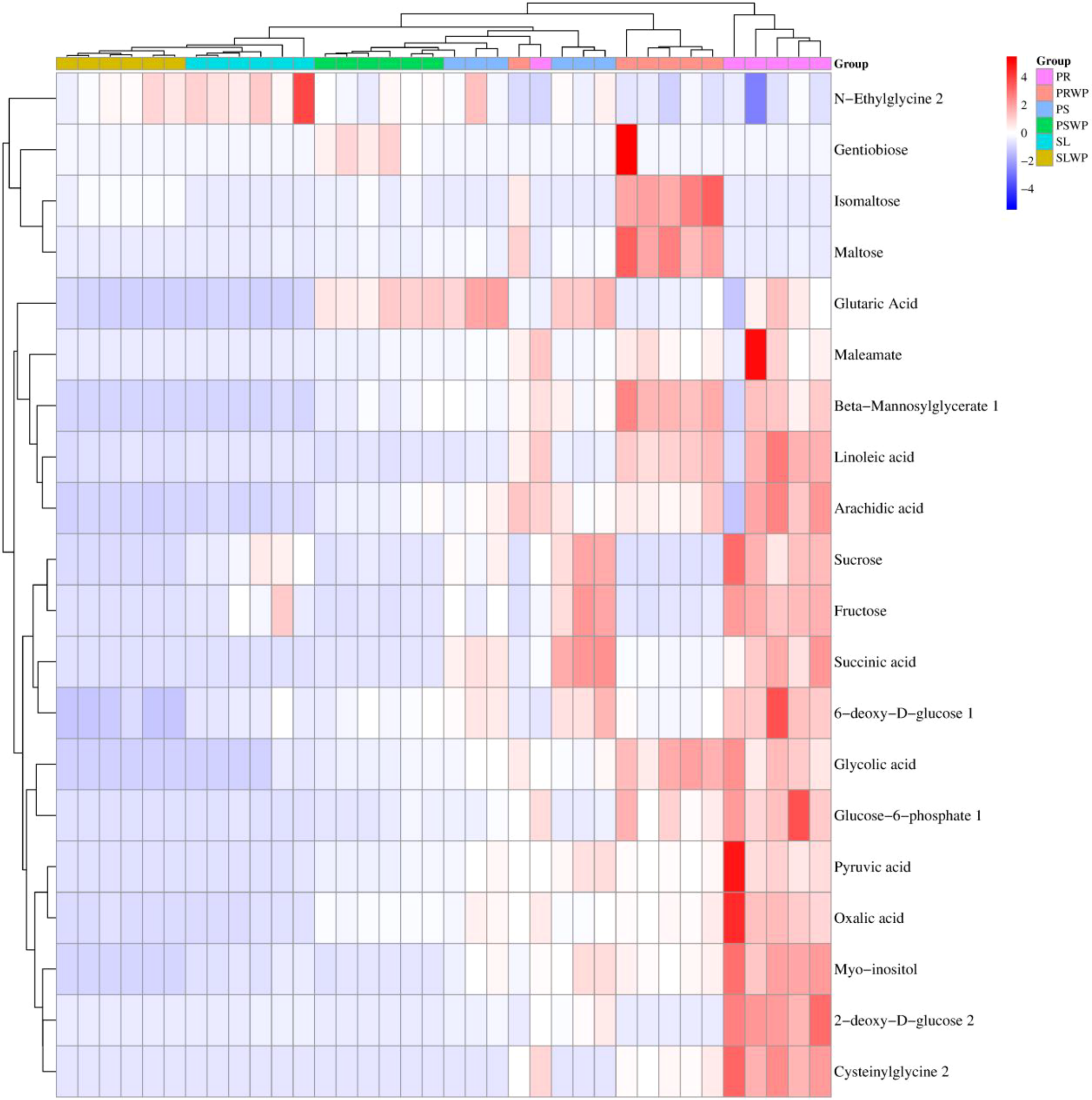
Heatmap of hierarchical clustering analysis of metabolites in tomato rhizosphere. The abscissa represents distinct sample groups while the ordinate denotes all metabolites, each column represents an individual biological replicate. The color blocks at various positions illustrate the relative expression levels of metabolites corresponding to those positions; red signifies high expression levels of substances, whereas blue indicates low expression levels.

Hierarchical clustering analysis (HCA) was performed on all detected metabolites across the six treatment groups, and the resulting heatmap is presented in **Supplementary Figure 2**. The clustering pattern showed that samples from the PR treatment formed a distinct cluster separate from all other treatment groups, indicating that the rhizosphere metabolite profile of PR was most dissimilar to those of SL and PS treatments. In contrast, samples from SLWP and SL were intermingled within the same cluster, suggesting high similarity in their metabolite expression patterns. Samples from PSWP clustered more closely with SLWP and SL than with PS, indicating that the metabolite profile of unplanted peat substrate resembled that of the soil groups more than that of the planted peat treatment. Based on the color distribution in the heatmap, the PR treatment exhibited the highest number of metabolites with increased abundance relative to other treatments; among these, Maleamate, Isomaltose, Pyruvic acid, and Gentiobiose showed the most pronounced increases (**Figure 2**). In the PS and PSWP treatments, metabolites with increased abundance were predominantly organic acids, alcohols, sugars, fatty acids, and polyketides. In contrast, the overall abundance of rhizosphere metabolites was lower in both SLWP and SL treatments compared to the organic substrate groups.

#### 3.3.2 Principal component analysis

**Figure 3:**
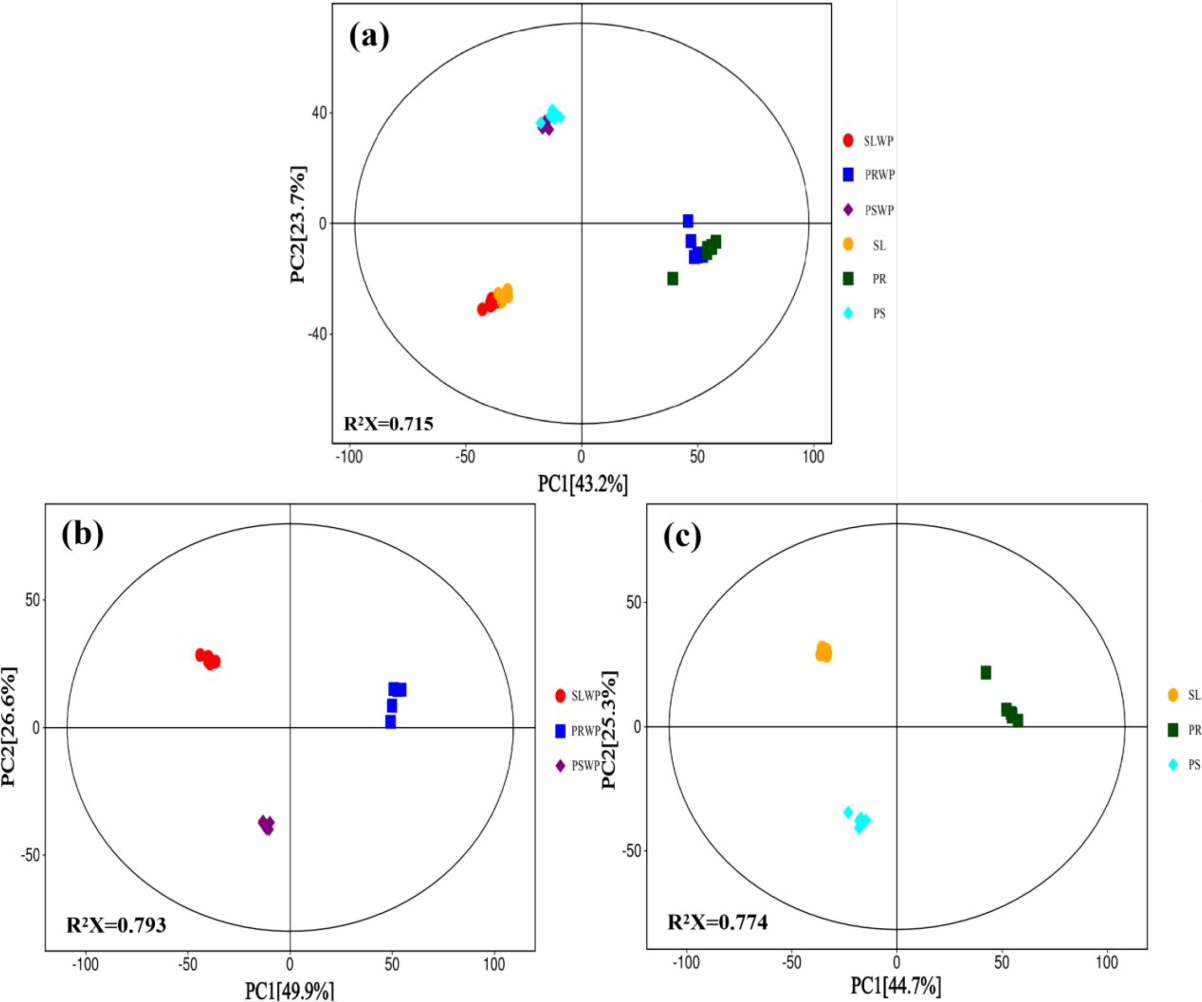
Principal component analysis of metabolites in tomato rhizosphere. Each point represents an individual biological replicate. **(a)** Principal component analysis of differential metabolites in tomatoes among six different treatments; **(b)** Principal component analysis of differential metabolites in various substrates of unplanted tomatoes; **(c)** Principal component analysis of differential metabolites in the rhizosphere of tomatoes grown on three different substrates.

After rigorous quality control, principal component analysis (PCA) in two dimensions was applied to reduce data dimensionality and visualize metabolic differences among the six soil and cultivation substrate treatments. As shown in the two-dimensional PCA score plot (**Figure 3a**), samples from the same treatment formed tightly clustered groups, indicating high intra-group metabolic homogeneity. In contrast, clear separations were observed among different treatments, reflecting distinct metabolic profiles. In the PCA plot, PSWP and PS samples clustered closely along the positive direction of PC2, while SLWP and SL treatments grouped in the negative region. The PR treatment and its control (PRWP) showed more pronounced separation compared to the other groups, with the PR samples exhibiting greater independence. Regarding variance explanation, the first two principal components collectively accounted for 66.9% of the total variance, with PC1 contributing 43.2% and PC2 explaining 23.7%. This indicates that both substrate type and the presence of a plant fundamentally reshape the rhizosphere metabolome.

### 3.4 Screening and analysis of differential metabolites in rhizosphere

#### 3.4.1 Differential metabolites in rhizosphere of tomato before and after cultivation

**Figure 4:**
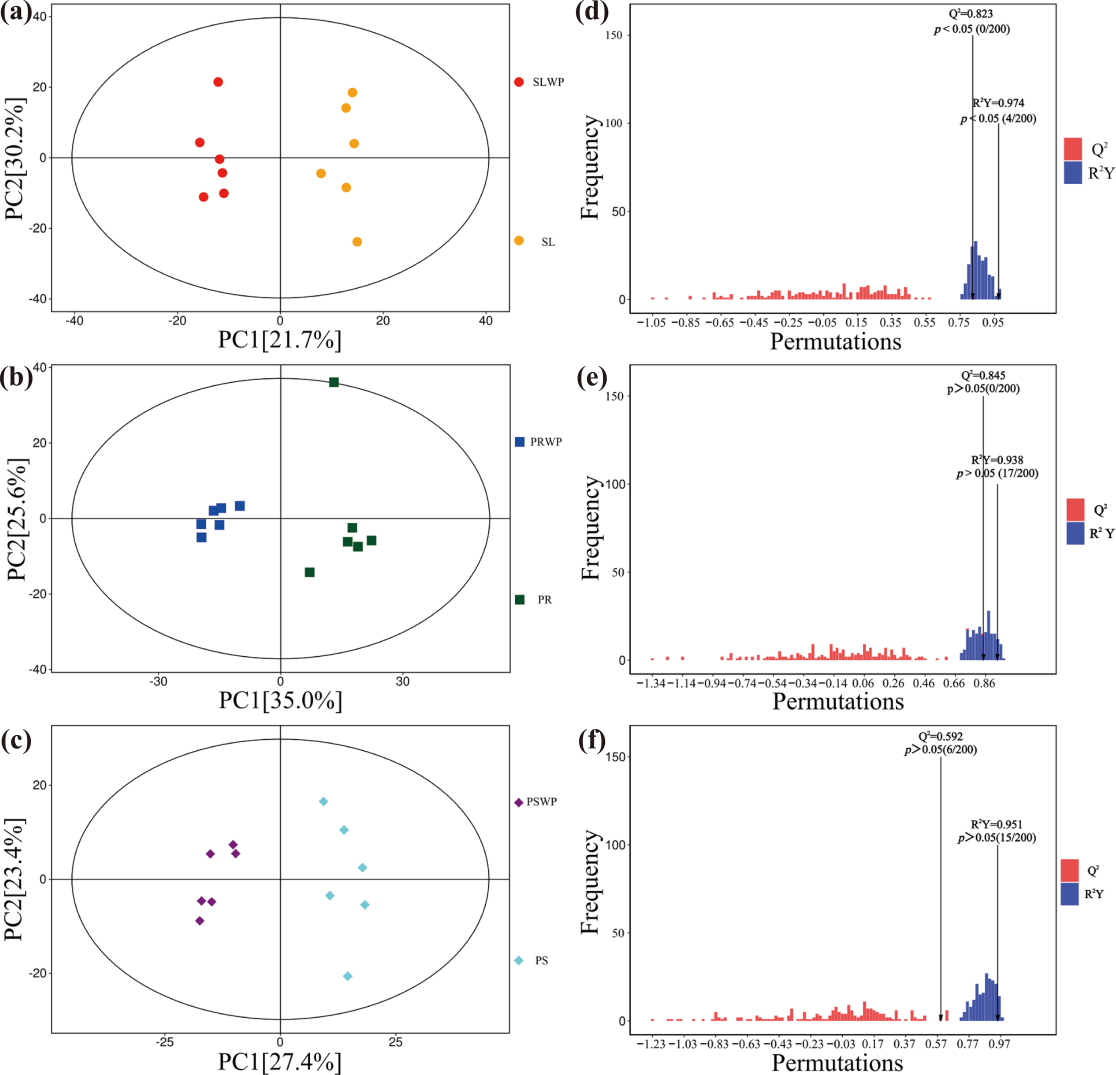
The OPLS-DA score plots and OPLS-DA permutation histograms of each control group. **(a)** OPLS-DA score plots of the SLWP and SL comparison groups; **(b)** OPLS-DA score plots of the PRWP and PR comparison groups; **(c)** OPLS-DA score plots of the PSWP and PS comparison groups; **(d)** OPLS-DA permutation histogram of the SLWP and SL comparison groups; **(e)** OPLS-DA permutation histogram of the PRWP and PR comparison groups; **(f)** OPLS-DA permutation histogram of the PSWP and PS comparison groups.

This section divided all the treatments into three control groups and compared the changes in metabolite components and expression levels of tomatoes after cultivation in soil (SLWP and SL), pinecone residue substrate (PRWP and PR), and peat substrate (PSWP and PS). The SLWP vs SL, PRWP vs PR and PSWP vs PS groups were analyzed and the score graphs were drawn respectively by using OPLS-DA (which can effectively eliminate the influence irrelevant to the study and thereby screen the differential metabolites). In this model, R^2^X and R^2^Y respectively represent the interpretation rates of the established model for the X and Y matrices, and Q^2^ indicates the predictive ability of the model. The Q^2^ of all comparison groups is higher than 0.592, indicating that the constructed model is appropriate. OPLS-DA models (**Figure 4**) effectively distinguished each substrate before (WP) and after tomato cultivation. The number of differential metabolites (VIP > 1, *p* < 0.05) varied by substrate: 141 in SL, 267 in PR, and 277 in PS (**Supplementary Table 5**). Among these, 129 (91.5%), 246 (92.1%), and 271 (97.8%) showed increased levels in SL, PR, and PS, respectively. Overall, more than 91% of the differential metabolites exhibited elevated abundance following tomato cultivation. This pattern indicates that tomato growth is associated with widespread increases in rhizosphere metabolite levels, pointing to an overall enhancement of metabolic activity rather than suppression.

**Figure 5:**
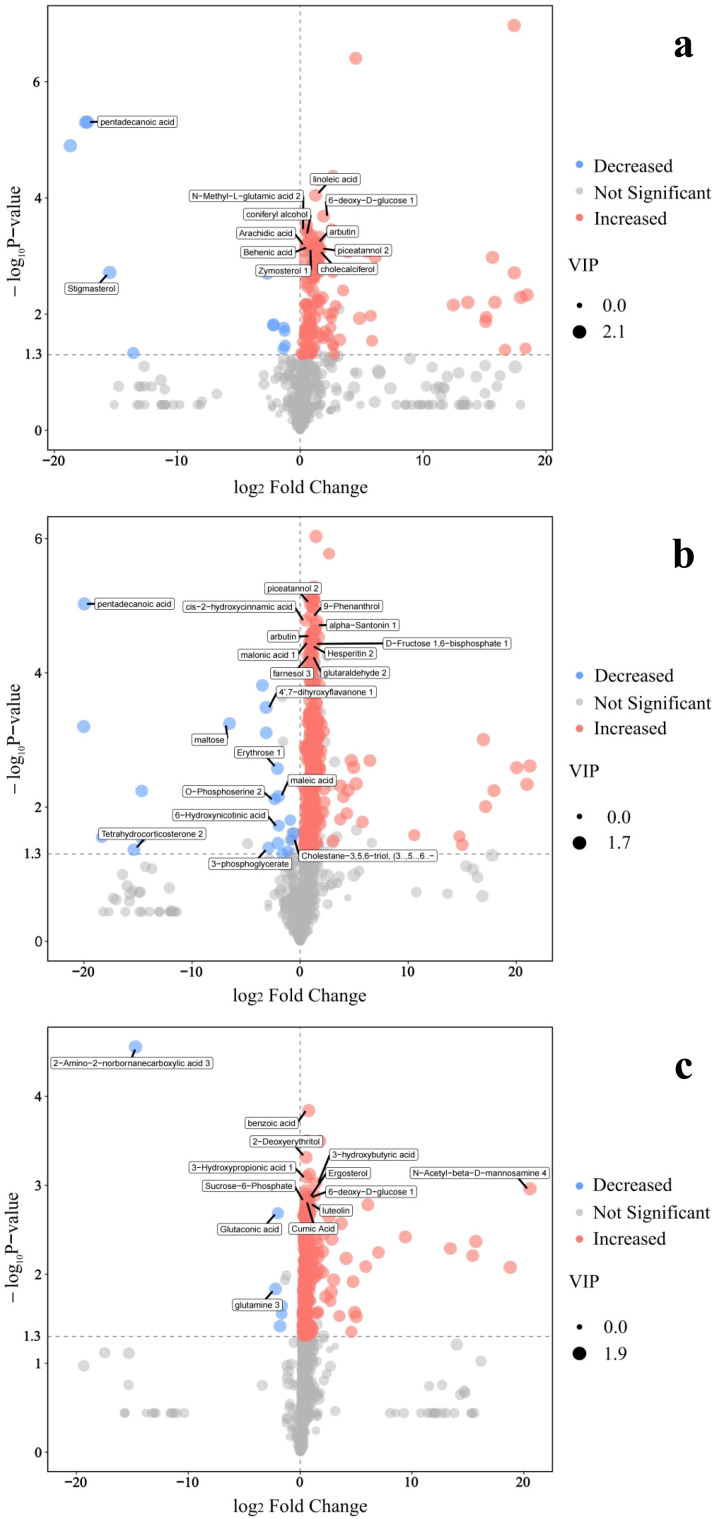
Volcanic plot of rhizosphere differential metabolites of tomato before and after cultivation. This figure illustrates the distribution of significantly up-regulated and down-regulated differential metabolites across three comparative groups: **(a)** SLWP vs. SL, **(b)** PRWP vs. PR, and **(c)** PSWP vs. PS. Metabolite annotation includes unidentified compounds and redundantly named entities; “analyte” prefixes denote unidentified metabolites, while “unknown” labels indicate non-unique nomenclature. Statistical analysis retained all differential metabolites irrespective of identification status.

Volcano plot (**Figure 5**) clearly illustrates the impact of tomato cultivation on the rhizosphere metabolome across different substrates. In the soil substrate (SLWP vs SL), 141 differential metabolites were identified. Among these, 129 showed significantly increased abundance, including linoleic acid, N-methyl-L-glutamic acid, 6-deoxy-D-glucose, coniferyl alcohol, arachidic acid, and arbutin. Only 12 metabolites decreased, primarily pentadecanoic acid and stigmasterol. In the pinecone residue substrate (PRWP vs PR), the number of differential metabolites increased to 267, with 246 displaying increased levels. Prominent among these were resveratrol, 9-phenolate, cis-2-hydroxycinnamic acid, arbutin, and malonic acid. Conversely, 21 metabolites showed decreased abundance, including maltose, erythritol, maleic acid, and tetrahydrocorticosterone. The most pronounced metabolic changes were observed in the peat substrate (PSWP vs PS), where 277 differential metabolites were detected. Of these, 271 exhibited significantly increased levels, such as benzoic acid, 2-deoxyerythritol, ergosterol, luteolin, and koleic acid. Only six metabolites decreased, including glutaric acid and glutamine.

**Figure 6:**
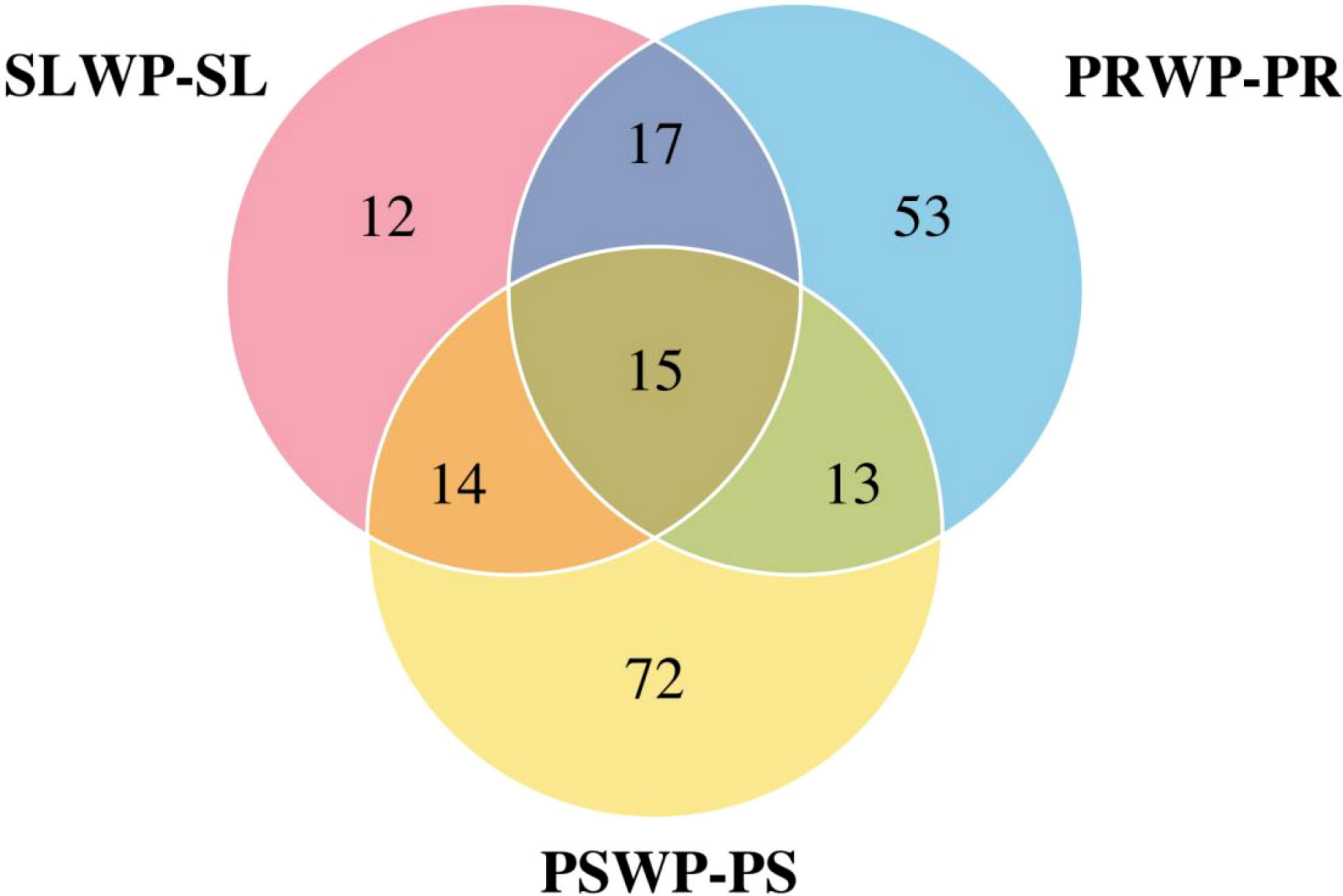
The rhizosphere differential metabolites Venn diagram of each comparison group before and after tomato cultivation. SLWP-SL means soil control vs soil, PRWP-PR means pine control vs pine, PSWP-PS means peat control vs peat.

After concealing the information of unknown substances and repeatedly named substances in the metabolites, the Venn diagram (**Figure 6**) was used to show the overlapping relationship of differential metabolites in the three control groups. It was found that the peat substrate (PS) had the most unique differential metabolites (72), which was much higher than that of soil (SL, 22) and pinecone residue substrate (PR, 43). This result indicates that the peat substrate induces the most significant substrate-specific metabolic reprogramming, and its rhizosphere metabolic response has higher uniqueness in composition.

**Figure 7:**
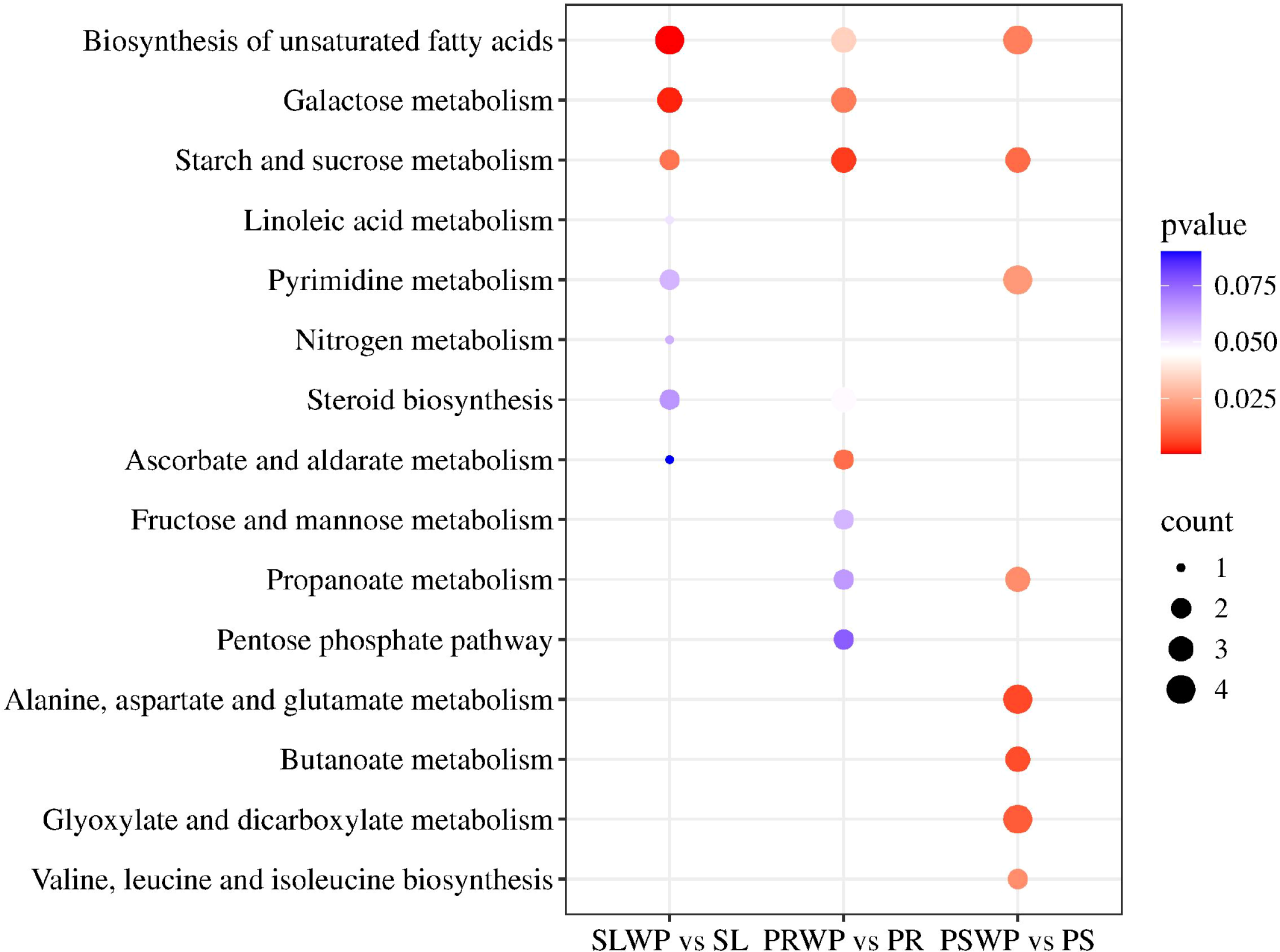
The KEGG enrichment of differential metabolites in the rhizosphere of tomatoes. *P* < 0.05 indicated that the pathway was significantly enriched. The size of the bubbles in this image and the values represented by the “count” legend reflect the number of differentially expressed metabolites (DEMs) that are enriched in this pathway.

KEGG pathway enrichment analysis (**Figure 7**) shows that unsaturated fatty acid biosynthesis and starch and sucrose metabolism are significantly enriched in all three substrates. The counts of differential metabolite are as follows: Unsaturated fatty acid biosynthesis (SLWP vs SL:4 Differentially expressed metabolites-DEMs, PRWP vs PR:3 DEMs, PSWP vs PS:4 DEMs); Starch and sucrose metabolism (SLWP vs SL:2 DEMs, PRWP vs PR:3 DEMs, PSWP vs PS:3 DEMs). Additionally, the SLWP vs SL comparison showed specific enrichment in Galactose metabolism (3 DEMs). The PRWP vs PR comparison was enriched in Galactose metabolism (3 DEMs) and Ascorbate and aldarate metabolism (2 DEMs). The PSWP vs PS comparison exhibited enrichment in multiple pathways: Pyrimidine metabolism (4 DEMs), Propanoate metabolism (3 DEMs), Alanine, aspartate and glutamate metabolism (4 DEMs), Butanoate metabolism (3 DEMs), Glyoxylate and dicarboxylate metabolism (4 DEMs), and Valine, leucine and isoleucine biosynthesis (3 DEMs).

#### 3.4.2 Effects of different cultivation media on differential metabolites in rhizosphere

To further elucidate the specific influence of cultivation substrates on the rhizosphere metabolic processes in tomatoes, this study categorized all treatments into two control groups: substrates without tomato cultivation (SLWP, PRWP, PSWP) and substrates with tomato cultivation (SL, PR, PS), enabling a comparative analysis of rhizosphere metabolomic profiles across different substrates under the presence and absence of plant growth. Principal component analysis (**Figure 3c**) revealed a clear separation of metabolic profiles among treatment groups, indicating that substrate type is a key determinant in shaping the rhizosphere metabolic environment of tomatoes. Specifically, the first principal component (PC1) effectively distinguished the PR treatment from SL and PS treatments, accounting for 44.7% of the total variance. The second principal component (PC2) further highlighted metabolic distinctions between the PS treatment and both SL and PR treatments, explaining an additional 25.3% of the variation. Differential metabolites were identified using one-way analysis of variance (ANOVA, *p* < 0.05), with unknown and repeatedly annotated compounds retained for statistical analysis. A total of 747 differential metabolites were detected in the unplanted control group, compared to 667 in the tomato-cultivated group. These findings demonstrate that the presence or absence of tomato cultivation significantly influences the composition and abundance of rhizosphere metabolites across different substrates, underscoring the active role of plants in modulating the rhizosphere metabolic landscape.

**Figure 8:**
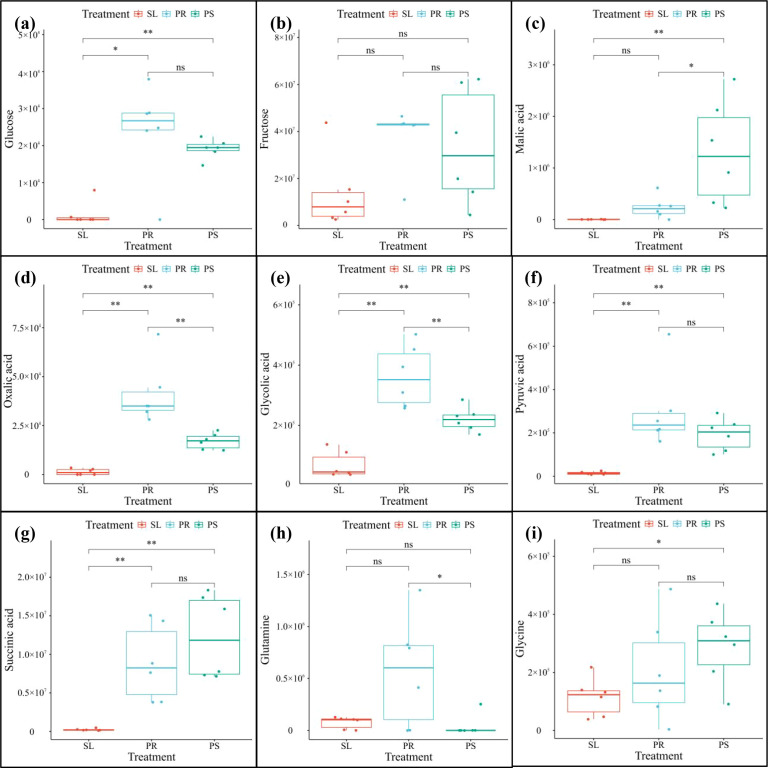
The effects of three cultivation substrates on the metabolites of tomato rhizosphere. **(a)** The influence of three cultivation substrates on Glucose content; **(b)** The influence of three cultivation substrates on Fructose content; **(c)** The influence of three cultivation substrates on Malic acid content; **(d)** The influence of three cultivation substrates on Oxalic acid content; **(e)** The influence of three cultivation substrates on Glycolic acid content; **(f)** The influence of three cultivation substrates on Pyruvic acid content; **(g)** The influence of three cultivation substrates on Succinic acid content; **(h)** The influence of three cultivation substrates on Glutamine content; **(i)** The influence of three cultivation substrates on Glycine content (ns: *p*≥0.05; *p < 0.05; **p < 0.01).

**Figure 8** shows the effects of three cultivation substrates on the metabolite contents in the tomato rhizosphere. The difference analysis indicates that compared with the SL treatment, the contents of *Glucose*, *Malic acid*, *Oxalic acid*, *Glycolic acid*, *Pyruvic acid*, *Succinic acid* and *Glycine* in the PS treatment were significantly increased; the contents of *Glucose*, *Oxalic acid*, *Glycolic acid*, *Pyruvic acid* and *Succinic acid* in the PR treatment were also significantly higher than those in the SL treatment. Moreover, in the comparison between the PS and PR treatments, the contents of *Oxalic acid*, *Glycolic acid*, *Pyruvic acid* and *Glutamine* in the PR treatment were significantly higher than those in the PS treatment, but the content of *Malic acid* was significantly lower than the latter.

## 4 Discussion

This study systematically investigated the associations between cultivation substrate properties, rhizosphere metabolic profiles, and tomato growth and fruit quality. The results show that the substrate type is not only a physical support but also a key ecological factor that actively participates in the “chemical dialogue” in the rhizosphere and drives the adaptive adjustment of plant metabolism (Yue et al., 2023). Compared with SL, PR and PS substrates have better physical structures (lower volumetric weight and better moisture content) and higher nutrient availability (especially available phosphorus, available potassium and exchangeable cations) (**Table 2**), thus creating a favorable microenvironment for root growth and nutrient absorption, microbial activities and metabolite exchange. Plant growth and yield data further confirm the positive effects of substrate treatment. Consistently, tomatoes grown in organic substrates, especially PS, showed enhanced growth and yield. The PS treatment was associated with the most pronounced increases in plant height, root length, organ biomass, and total fruit yield. These improvements point to more effective root system development and enhanced allocation of photosynthetic assimilates to reproductive organs under peat cultivation. Fruit quality was also modulated by substrate type. Both PR and PS treatments increased sugar–acid ratio, soluble solids, and vitamin C content relative to SL. Notably, PS treatment yielded the highest vitamin C levels, suggesting that substrate properties can influence fruit nutritional and sensory quality (Guo et al., 2022). The observed differences in fruit quality and overall plant performance are likely rooted in the substrate’s fundamental chemical characteristics. The physicochemical properties of a substrate, including its pH, electrical conductivity (EC), and organic matter content, directly govern the availability of nutrients. For instance, the higher levels of available and exchangeable potassium (K^+^) found in the PS and PR substrates (**Table 2**) are particularly significant. Potassium is a key regulator of plant physiology. It activates enzymes involved in chlorophyll synthesis and photosynthetic carbon assimilation, thereby enhancing photosynthetic efficiency and promoting coordinated shoot and root development (Lebaudy et al., 2008). Beyond these direct physiological roles, nutrient availability profoundly shapes the plant’s metabolic profile. The synergistic enhancement of the above physiological processes not only helps to improve the lodging resistance and drought resistance of plants, but ultimately promotes yield formation. This suggests that organic substrates may comprehensively optimize fruit quality by synergistically strengthening the antioxidant metabolism and secondary metabolism pathways of plants (Li et al., 2025). More specifically, the ample potassium in PS may directly stimulate the biosynthesis of ascorbic acid (vitamin C), as potassium is a known cofactor for enzymes in this pathway. Similarly, the balance of nitrogen forms (ammonium vs. nitrate) can dictate the profile of amino acids and organic acids synthesized in the plant and subsequently exuded into the rhizosphere. Thus, the chemical composition of the substrate does not merely serve as a nutrient reservoir; it acts as a signal that primes specific metabolic pathways, leading to the distinct metabolic signatures observed in the rhizosphere.

Non-targeted metabolomics analysis revealed that the rhizosphere metabolic profile of tomato exhibited a highly specific response to the type of cultivation substrate (Zhao et al., [[NoYear]]). In principal component analysis, PC1 (44.7%) clearly separated the PR treatment from SL and PS treatments (**Figure 3c**), indicating that PR induced a distinct metabolic pattern, potentially attributable to its unique organic matter composition and degradation products. Further analysis of differential metabolites demonstrated that the PS treatment yielded the highest number of differentially accumulated metabolites (n=277) and exhibited the highest metabolic diversity. Notably, PS was the only treatment in which defensive secondary metabolites, such as “Alkaloids and derivatives” and “Lignans, neolignans and related compounds”, were detected (**Figures 1b–d**). KEGG pathway enrichment analysis further confirmed that the PS treatment specifically activated propanoate metabolism, glyoxylate-dicarboxylate metabolism, and several amino acid biosynthesis pathways (**Figure 7**), indicating that PS triggers a more complex and functional metabolic reprogramming, one that not only enhances fundamental energy and material metabolism but also systematically orchestrates secondary metabolic pathways associated with stress resistance and defense (Chen et al., 2022). In contrast, the metabolic response under SL was relatively conservative, characterized by the lowest number of differential metabolites (n=141) and the absence of specific defense-related metabolites. These findings imply that, under the constraints of suboptimal physicochemical properties in continuously cropped soils and stabilized microbial communities, the capacity of tomato roots to actively modulate their rhizosphere chemical environment may be limited (Jiang et al., 2024). Closer examination of metabolites with increased levels revealed substrate-specific adaptive strategies. In the PS treatment, the concentrations of oxalic acid, glycolic acid, pyruvate, and succinic acid were significantly higher than those in SL (**Figure 8**). This pattern suggests that under mild substrate acidification (pH = 5.00), tomato plants do not merely tolerate the condition but actively enhance the secretion of organic acids—particularly oxalic acid. Oxalic acid plays a critical role in regulating intracellular calcium homeostasis and alleviating aluminum toxicity through the formation of calcium oxalate crystals (Alarcon-Poblete et al., 2020; Topcuoğlu, 2022). The concurrent increase in glycolic acid and succinic acid suggests enhanced organic acid metabolism, which may reflect shifts in photorespiratory carbon flux or tricarboxylic acid (TCA) cycle activity under PS conditions (Zhao et al., 2022; Shi et al., 2023; Zhuang et al., 2023). Glycolic acid is the primary substrate of photorespiration, and its accumulation could indicate increased Rubisco oxygenase activity or altered photorespiratory carbon flow (Yao et al., 2022; Siqueira et al., 2023). Meanwhile, the observed elevation of succinic acid—a key intermediate of both the TCA cycle and the glyoxylate cycle—points to potential activation of this alternative carbon-conserving pathway (Lü et al., 2019; Abbey et al., 2022). The glyoxylate cycle enables plants to convert lipids into carbohydrates and may help maintain metabolic flexibility under environmental constraints (Zhang and Bryant, 2015; Kleczkowski and Igamberdiev, 2024). Such metabolic flux remodeling represents an efficient strategy for plants to adapt to dynamic changes in the rhizosphere chemical environment (Ofoe et al., 2023). Moreover, “Lipids and lipid molecules” constituted the most abundant metabolite class across all treatments (**Figure 1**), with particularly pronounced up-regulation observed in the PR treatment. Lipids serve not only as fundamental structural components of cellular membranes but also as precursors for defense signaling molecules, including *Jasmonic acid (*Mandavi et al., 2025). Their widespread up-regulation suggests that different substrates trigger substantial membrane remodeling and lipid-mediated signal transduction processes, which are essential for preserving root cell integrity, perceiving environmental cues, and initiating downstream stress responses (Liang et al., 2023; Maryam et al., 2025). Furthermore, the differential accumulation of glycine in PS and glutamine in PR (**Figure 8**) reflects distinct nitrogen availability, likely driven by enhanced ammonium accumulation in PS (**Table 2**), as well as variations in microbial nitrogen transformations across substrates. These variations may influence the nitrogen assimilation strategies and patterns of amino acid exudation in tomato roots, thereby contributing to feedback regulation of the rhizosphere nitrogen cycle (Sun et al., 2023). Finally, it is important to acknowledge that the rhizosphere metabolome represents a composite signature derived from both root exudation and microbial activity. The observed changes—especially in PR and PS, which harbor more diverse microbial communities (Grunert et al., 2016)—likely reflect complex interactions between plant-driven responses and microbial processing of root-derived compounds. Our bulk metabolomics approach cannot disentangle these contributions, a limitation that should be considered when interpreting results. Future studies integrating metagenomics, metatranscriptomics, or stable isotope probing could provide deeper insight into the specific roles of microbial metabolism in shaping substrate-specific rhizosphere environments and their subsequent effects on plant performance.

This study revealed a key finding: tomato cultivation reduced the number of differential rhizosphere metabolites across substrates from 747 in the unplanted control to 667. This decrease reflects not a decline in metabolic activity, but rather a pronounced remodeling of the rhizosphere microenvironment by tomato roots, strongly supporting the “plant-dominated” rhizosphere shaping theory (Zuluaga et al., 2021; Yue et al., 2023). Despite significant inherent chemical heterogeneity among the unplanted substrates, the tomato root system actively drives the rhizosphere metabolome toward a more convergent and growth-favorable state through root exudation and microbial recruitment (Zuluaga et al., 2021). This plant-induced convergence partially masks the background differences of the substrates and underscores the proactive regulatory role of plants in shaping the rhizosphere. The 667 retained differential metabolites reflect the boundaries set by the inherent characteristics of different substrates (such as the acidity of PS and the nutrient composition of PR) for plant metabolic regulation, as well as the specific metabolic responses triggered by tomatoes to adapt to these specific conditions (as shown in **Figure 6**, PS has the most unique differential metabolites). It is precisely this metabolic regulation model that coexists with universal convergence and specific adaptation that jointly constitutes the microscopic basis for tomatoes to achieve excellent agronomic traits in different facility cultivation substrates (**Tables 3**, **5**), and further forms a metabolic spectrum of functional differentiation such as PS tending toward “defense enhancement type” and PR tending toward “lipid activation type”. The core contribution of this study lies in the effective connection between the changes in the rhizosphere metabolome and the observable plant phenotypes. The most active and diverse metabolic reprogramming under PS treatment - especially the activation of defensive secondary metabolic pathways - provided a direct biochemical basis for a significant 50.19% increase in vitamin C content in its fruits. Vitamin C (ascorbic acid), as a core antioxidant molecule in plants, its biosynthesis is closely related to multiple secondary metabolic and stress response pathways (Paciolla et al., 2019; Sami et al., 2024). Meanwhile, the active organic acid metabolism, enhanced lipid and energy metabolism jointly promoted the vitality of the root system and the efficiency of nutrient absorption (Wen et al., 2020)(**Table 4**), providing sufficient material and energy support for the accumulation of aboveground biomass and the increase in yield (PS increased by 80.08%) (Ceccarelli et al., 2021). The optimization of the sugar-acid ratio in fruits may stem from the redistribution of carbon metabolic flows within the fruits. Therefore, PS does not merely promote plant growth through nutrient supply, but rather triggers a systematic rhizosphere metabolic process through its specific physicochemical properties (such as mild acidity and high nutrient availability), simultaneously optimizing the plant’s stress resistance, nutrient absorption and fruit quality formation.

In conclusion, this study has revealed the cascading relationship among “substrate characteristics - rhizosphere metabolic profile - plant phenotypes” at the metabolomics level. Organic substrates, especially peat substrates, induce extensive and specific metabolic reprogramming in the rhizosphere of tomatoes by providing specific physicochemical cues, characterized by enhanced basal metabolism and systematic activation of defensive secondary metabolism. This metabolic adjustment strategy significantly enhanced tomatoes’ adaptability to the rhizosphere environment, ultimately translating into advantages in growth and quality. This study provides a theoretical basis for the precise selection and optimization of cultivation substrates for facility tomatoes. Future research can further integrate metagenomic or metatrtomic techniques to analyze the relative proportion of plant root secretion and microbial metabolic contribution in the observed metabolic changes, and verify the specific functions of key metabolites (such as specific alkaloids and oxalic acid) in mediating plant-microbial interactions, thereby achieving more precise and targeted regulation of the rhizosphere microenvironment.

A methodological consideration in this study is the unequal total nutrient supply across treatments, resulting from differences in the native nutrient contents of the substrates despite the application of equal amounts of controlled-release fertilizer. This confounding factor means that the observed phenotypic and metabolic differences cannot be attributed solely to substrate-induced reprogramming, as direct nutrient effects may also contribute. However, several lines of evidence suggest that substrate properties beyond mere nutrient content play a significant role: (i) the distinct metabolic profiles between PR and PS, which both have high nutrient availability but differ in organic matter composition; (ii) the activation of specific secondary metabolic pathways (e.g., alkaloid biosynthesis in PS) that are not directly linked to macronutrient levels; and (iii) the strong correlations between physical properties (e.g., bulk density, moisture content) and metabolic patterns. Nevertheless, we acknowledge that future experiments with nutrient-matched substrates or factorial designs are necessary to disentangle nutrient effects from substrate-mediated metabolic regulation. Despite this limitation, our findings provide valuable insights into the complex interplay between substrate characteristics and rhizosphere metabolism under realistic horticultural conditions.

## 5 Conclusion

This study employs GC-MS-based untargeted metabolomics analysis to demonstrate that the type of cultivation substrate is strongly associated with distinct rhizosphere metabolic profiles and tomato performance. Compared with facility soil, organic substrates (particularly peat) exhibit superior physicochemical properties and are associated with enhanced root metabolic activity. Metabolomic data reveal that different substrates induce distinct metabolic patterns: peat substrate is characterized by up-regulation of defense-related metabolites (e.g., alkaloids and lignans) and organic acids, whereas pinecone residue substrate predominantly activates lipid metabolic pathways. These metabolic alterations are correlated with plant phenotypic performance: under peat treatment, the observed metabolic reprogramming coincides with higher yield (+80.08%) and fruit vitamin C content (+50.19%). While these associations suggest that organic substrates may promote crop growth partly through modulation of the rhizosphere metabolic microenvironment, further studies are needed to establish causal relationships and to disentangle the contributions of direct nutrient effects, plant physiology, and microbial processes.

## Data Availability

The original contributions presented in the study are included in the article/**Supplementary Material**. Further inquiries can be directed to the corresponding authors.
