# Correction: Substrate-driven reprogramming of the rhizosphere metabolome underlies enhanced tomato growth and quality in soilless cultivation

**DOI:** 10.3389/fpls.2026.1850846

**Published:** 2026-04-16

**Authors:** Yu Chen, Yuyuan Chai, Xi Chen, Jing Shi

**Affiliations:** 1 College of Resources and Environment, Yunnan Agricultural University, Kunming, China; 2 Key Laboratory for Improving Quality and Productivity of Arable Land of Yunnan Province, College of Resources and Environment, Yunnan Agricultural University, Kunming, Yunnan, China; 3 Kunming Changshui International Airport Power Energy Department, Kunming, China

**Keywords:** culture medium, metabolomics, peat substrate, pinecone residue substrate, rhizosphere metabolites

The **Author contributions** statement was erroneously given as “YC: Writing – original draft, Writing – review & editing. YYC: Writing – original draft. XC: Validation, Writing – review & editing. JS: Funding acquisition, Writing – review & editing”. The correct **Author contributions** statement is “YC: Writing – original draft, Writing – review & editing. YYC: Writing – original draft, Data curation. XC: Validation, Writing – review & editing. JS: Funding acquisition, Writing – review & editing”.

Author Yu Chen was erroneously assigned as corresponding author. The correct corresponding author is “Jing Shi.”

Author “Yuyuan Chai” was erroneously omitted as equal contributing author.

The original version of this article has been updated.

