# Supplementary material for: Substrate-driven reprogramming of the rhizosphere metabolome underlies enhanced tomato growth and quality in soilless cultivation
Source: Front Plant Sci. 2026 Apr 1;17:1783719. doi: 10.3389/fpls.2026.1783719 (PMC13079676; doi:10.3389/fpls.2026.1783719)
Supplement: Supplementary file 1 [file DataSheet1.docx]

**Table S1 Test Design**

| Experiment number | Treatment | | The amount of cultivation substrate per pot | Repeats of each treatment |
| --- | --- | --- | --- | --- |
|  | Culture substrate | Plant |  |  |
| SLWP | Soil | / | 0.02m^3^ | Six times |
| PRWP | pinecone residue substrate | / |  |  |
| PSWP | peat substrate | / |  |  |
| SL | Soil | Tomato (22B) |  | Thirty-four times |
| PR | pinecone residue substrate | Tomato (22B) |  |  |
| PS | peat substrate | Tomato (22B) |  |  |

**Note:** The ' / ' in the table indicates that no plants have been planted.

**Table S2 List of experimental reagents**

| Name | CAS | Purity | Brand |
| --- | --- | --- | --- |
| Methanol | 67-56-1 | HPLC | CNW Technologies |
| Chloroform | 67-66-3 | HPLC | Adamas |
| Pyridine | 110-86-1 | HPLC | Adamas |
| Methoxyaminatio hydrochloride | 593-56-6 | AR | TCI |
| Adonitol | 488-81-3 | ≥99% | SIGMA |
| BSTFA（with 1% TMCS, v/v） | -- | -- | REGIS Technologies |
| Saturated fatty acid methyl ester（FAMEs） | -- | -- | Dr. Ehrenstorfer |
| Pure water（ddH_2_O） | -- | -- | From the pure water device |
| Ethyl Acetate | 141-78-6 | HPLC | CNW Technologies |

**Table S3 Experimental Instruments Used for Untargeted Metabolomics Detection**

| Experimental apparatus | Model | Brand name |
| --- | --- | --- |
| Gas chromatograph | GC-2030 | Shimazu |
| Mass spectrometer | QP2020 NX | Shimazu |
| Chromatographic column | DB-5MS(30 m×250 μm×0.25 μm) | Agilent |
| Centrifuge | Heraeus Fresco17 | Thermo Fisher Scientific |
| Ultralow temperature freezer | Forma 900 series | Thermo Fisher Scientific |
| Analytical balance | BSA124S-CW | Sartorius |
| Grinder | JXFSTPRP-24 | Shanghai Jingxin Technology Co., Ltd. |
| Ultrasonic apparatus | YM-080S | Shenzhen Fang Ao Microelectronics Co., Ltd. |
| Drying oven | DHG-9023A | Shanghai Yiheng Scientific Instrument Co., Ltd. |
| Vacuum Dryer | LNG-T98 | Taicang Huamei Biochemical Instrument Factory |

**Table S4 Instrument parameters**

| Project | Parameters |
| --- | --- |
| Sample Volume | 1 μL |
| Front Inlet Mode | Splitless Mode |
| Front Inlet Septum Purge Flow | 3 mL min^−1^ |
| Carrier Gas | Helium |
| Column | DB-5MS（30 m×250 μm×0.25 μm） |
| Column Flow | 1 mL min^−1^ |
| Oven Temperature Ramp | 50 °C hold on 1 min, raised to 310 °C at a rate of 8 °C min^−1^, hold on 11.5 min |
| Front Injection Temperature | 280 °C |
| Transfer Line Temperature | 280 °C |
| Ion Source Temperature | 200 °C |
| Electron Energy | -70 eV |
| Mass Range | m/z:50-500 |
| Acquisition Rate | 12.5 spectra per second |
| Solvent Delay | 7.2 min |

**Table S5 Statistics of differential metabolites before and after cultivation of tomatoes**

| Comparison groups | Total number of detected substances | Number of differential substances | The number of metabolites with Increased levels | The number of metabolites with Decreased levels |
| --- | --- | --- | --- | --- |
| SLWP and SL | 848 | 141 (16.63%) | 129 (91.49%) | 12 (8.51%) |
| PRWP and PR | 848 | 267 (31.49%) | 246 (92.13%) | 21 (7.87%) |
| PSWP and PS | 848 | 277 (32.67%) | 271 (97.83%) | 6 (2.17%) |

**Note:** The proportion of the different substances = the quantity of the different substances / the total quantity of substances detected; The proportion of the increased (decreased) level of different substances = the quantity of the increased (decreased) level of different substances / the total quantity of different substances. The statistical results do not include unknown substances and substances with duplicate names.

Fig. S1 All QC sample TIC plots


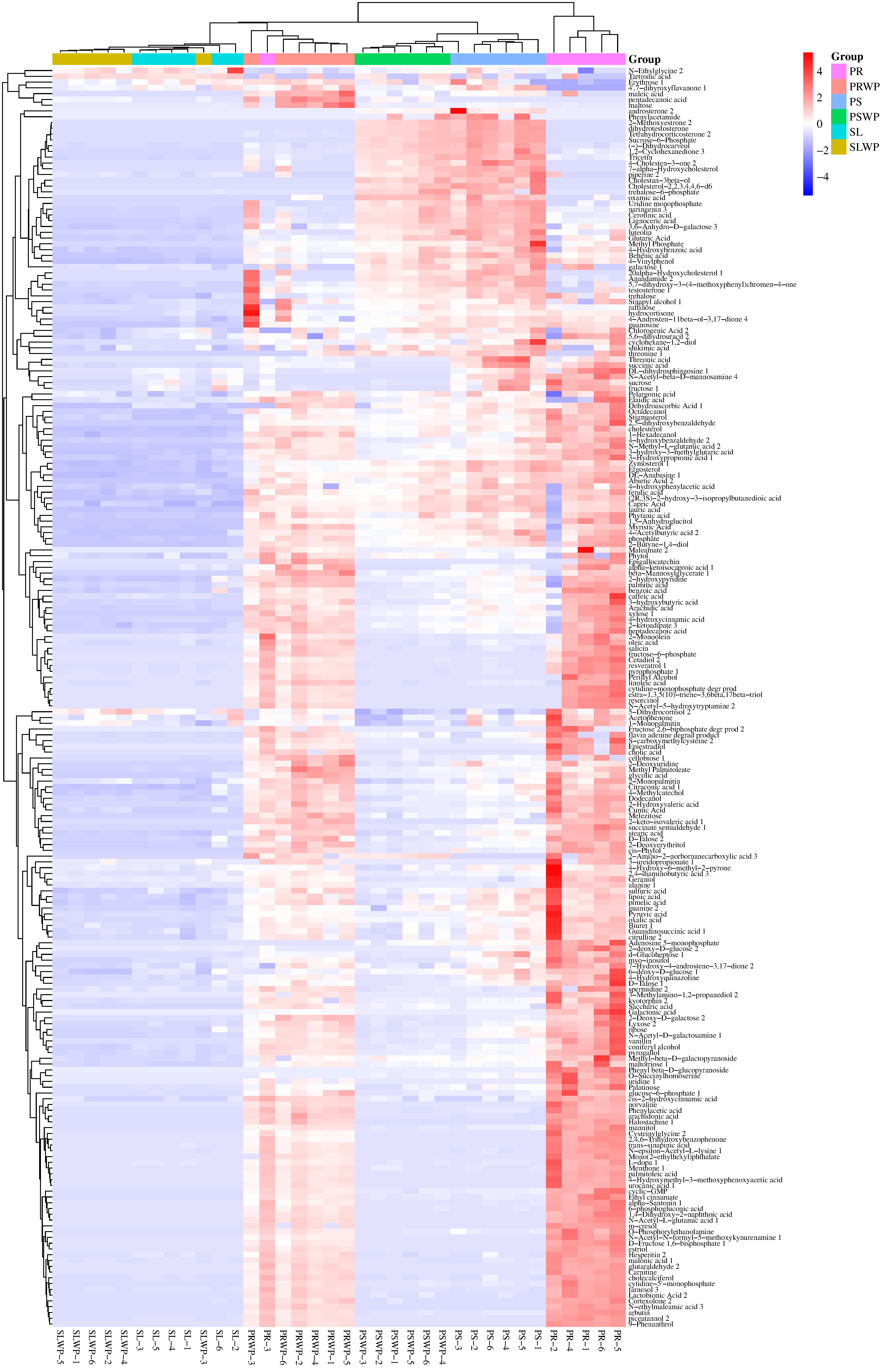


Fig. S2 Heatmap of Hierarchical Clustering Analysis of Metabolites in Tomato Rhizosphere
